# The Potential Trajectory of Carbapenem-Resistant *Enterobacteriaceae*, an Emerging Threat to Health-Care Facilities, and the Impact of the Centers for Disease Control and Prevention Toolkit

**DOI:** 10.1093/aje/kwv299

**Published:** 2016-02-08

**Authors:** Bruce Y. Lee, Sarah M. Bartsch, Kim F. Wong, James A. McKinnell, Rachel B. Slayton, Loren G. Miller, Chenghua Cao, Diane S. Kim, Alexander J. Kallen, John A. Jernigan, Susan S. Huang

**Keywords:** carbapenem-resistant *Enterobacteriaceae*, control measures, coordinated responses, regional spread, surveillance

## Abstract

Carbapenem-resistant *Enterobacteriaceae* (CRE), a group of pathogens resistant to most antibiotics and associated with high mortality, are a rising emerging public health threat. Current approaches to infection control and prevention have not been adequate to prevent spread. An important but unproven approach is to have hospitals in a region coordinate surveillance and infection control measures. Using our Regional Healthcare Ecosystem Analyst (RHEA) simulation model and detailed Orange County, California, patient-level data on adult inpatient hospital and nursing home admissions (2011–2012), we simulated the spread of CRE throughout Orange County health-care facilities under 3 scenarios: no specific control measures, facility-level infection control efforts (uncoordinated control measures), and a coordinated regional effort. Aggressive uncoordinated and coordinated approaches were highly similar, averting 2,976 and 2,789 CRE transmission events, respectively (72.2% and 77.0% of transmission events), by year 5. With moderate control measures, coordinated regional control resulted in 21.3% more averted cases (*n* = 408) than did uncoordinated control at year 5. Our model suggests that without increased infection control approaches, CRE would become endemic in nearly all Orange County health-care facilities within 10 years. While implementing the interventions in the Centers for Disease Control and Prevention's CRE toolkit would not completely stop the spread of CRE, it would cut its spread substantially, by half.

Carbapenem-resistant *Enterobacteriaceae* (CRE) are considered an urgent public health threat by the Centers for Disease Control and Prevention (CDC) (1). Few treatment options exist for CRE infection, which can result in high mortality. The emergence of CRE across the United States has suggested that existing approaches to infection control and prevention may not be adequate to control the spread of CRE. CRE have been steadily increasing in prevalence over the past decade in many US regions. In 2011, 4.2% of *Enterobacteriaceae* were CRE, up from the 1.2% reported in 2001 (2, 3). CRE detection increased more than 5-fold between 2008 and 2012 (4). Within the first 6 months of 2012, 3.9% of acute-care hospitals and 17.8% of long-term acute-care hospitals (LTACs) reported at least 1 CRE infection (2). Additionally, as of January 2015, CRE has been confirmed in 48 states (5). These data strongly suggest that CRE will eventually become widespread if new tactics in controlling the spread of CRE are not adopted.

Some regions of the United States have experienced a particularly high burden or rapid spread of CRE (6). In regions with high CRE burden, the CDC has recommended (i.e., in the 2012 CRE toolkit) that health-care facilities in the region coordinate CRE surveillance and control measures (7). Previous studies have demonstrated the benefits of regional coordination of control measures for endemic pathogens such as methicillin-resistant *Staphylococcus aureus* (MRSA) and vancomycin-resistant enterococci (8–10). However, the dynamics of emerging pathogens are different from those of endemic pathogens, and CRE have specific characteristics that may mean that their patterns of spread are different in varying scenarios and settings, which leads to several key questions. First, if current trends persist, what will CRE prevalence in health-care facilities be in the coming years? Second, will existing approaches be adequate (i.e., each health-care facility acting independently in CRE control)? Third, what are the added benefits of regional implementation of the CDC's 2012 CRE toolkit (7), and how should the regional interventions be implemented (e.g., at what thresholds should aggressive control measures be employed)?

## METHODS

### The RHEA model

We used our previously described Regional Healthcare Ecosystem Analyst (RHEA) software (11) to generate an agent-based model of CRE for Orange County, California, which included detailed representations of all 28 acute-care hospitals serving adult patients (including 5 LTACs) and 74 nursing homes and the patients moving among these facilities and the community. We used the Orange County agent-based model to simulate CRE transmission and evaluate regional interventions. Table 1 shows key model inputs. Our model drew from detailed 2011–2012 Orange County patient-level data for adult inpatient hospital and nursing home admissions (12, 13).

**Table 1.** Key Input Parameters, Values, and Sources Used in the RHEA Model to Simulate the Spread of Carbapenem-Resistant *Enterobacteriaceae* in Orange County, California

Parameter	Type of Health-Care Facility						Source (Reference No.)
	Acute-Care Hospitals		Long-Term Acute-Care Hospitals		Nursing Homes		
	Median	Range	Median	Range	Median	Range	
*Facility Characteristics*							
Daily capacity^a^	125	14–356	69	26–100	97	27–277	12, 13
Annual no. of adult admissions	7,588	779–24,998	1,076	371–2,541	345	35–1,554	12, 13
Mean length of stay, days	5.4	4.2–6.9	25.7	12.3–34.2	107	36–362	12, 13
No. of discharges to community	3,552	277–15,604	350	159–1,785	245	20–1,462	12, 13
No. of direct transfers to hospitals	309	34–1,373	64	1–295	33	23–152	12, 13
No. of direct transfers to nursing homes	931	160–2,253	163	14–449	60	0–480	12, 13
No. of readmissions	1,952	276–6,318	323	186–1,005	318	11–1,317	12, 13
Time to readmission, days	91.6	76.1–104.4	72.4	41.4–113.6	44	1–365	12, 13
No. of temporary discharges to hospitals					65	0–192	13
Length of temporary stay, days					6	0–14	13
*CRE Parameters*							
Targeted point prevalence at year 7 from CRE emergence in Orange County, %			25		8		23–27
Ratio of carriers to clinical isolates	8:1		8:1		8:1		16, 28, 33^b^
Transmission coefficient	ICUs: 0.00025095		0.00467996	0.00411885–0.00834316	0.000057895	0–0.00053513	—^c^
	General wards: 0.0001673						
Increased risk of readmission for CRE carriers on discharge, %	80		80		80		22^d^
Persistent carriers (those who remain colonized), %	30		30		30		34, 35
Loss rate for CRE carriage at 12 months, %^e^	50		50		50		36
Sensitivity of single rectal swab, %	70		70				17
Screening test sensitivity, %	91	85–92	91	85–92			18–20
Screening test specificity, %	94	89–97	94	89–97			18–20
Test turnaround time, days	1		1				37

Abbreviations: CRE, carbapenem-resistant *Enterobacteriaceae*; ICU, intensive care unit; RHEA, Regional Healthcare Ecosystem Analyst.

^a^ The average daily number of patients in a facility.

^b^ Also personal communication with Dr. Michael Lin (Rush University, Chicago, Illinois) on November 3, 2014.

^c^ Parameterized by model.

^d^ Also personal communication with Dr. Dawn Terashita (Los Angeles County Department of Public Health, Los Angeles, California) on January 12, 2013.

^e^ Assumes a linear loss for the remaining 70% of carriers who experience loss of CRE carriage.

Briefly, the model represents each patient with a computational agent, which on a given day can either carry or not carry CRE (14). On each simulated day, thousands of these agents move from the community or other health-care facilities into the various health-care facilities in Orange County. Each virtual health-care facility has a number of virtual beds, based on its actual bed count. Acute-care facilities consist of multiple wards (general wards and intensive care units), while nursing homes consist of a single large ward, representing the high degree of social interaction among residents. Once a patient is admitted to a facility, a probability draw determines which of that facility's wards/units the patient will enter, and a draw from a facility and unit-/ward-specific length-of-stay distribution determines how long the patient will remain in the ward/unit and facility. CRE carriers draw from a CRE-specific length-of-stay distribution (on average, 7.6 days longer than noncarriers) generated from data on vancomycin-resistant enterococci carriage in Orange County (12). If transferring directly to an acute-care hospital, a patient from an LTAC would be admitted to an intensive care unit 50% of the time and a resident from a nursing home would be admitted to the intensive care unit 20% of the time (15), to represent patients requiring mechanical ventilation or similar intensive care.

Each day, within each ward/unit, patients mix homogeneously, and CRE carriers can transmit CRE to noncarriers, based on a ward- and facility-specific transmission coefficient (β): β × susceptible patients × infectious CRE patients. Once the patient's stay ends, the patient leaves the facility and has probabilities of returning to the community, directly transferring to another Orange County facility, or returning to the community for a period of time before being readmitted to the same or another facility. During a nursing home stay, a resident can experience a brief hospitalization during which his/her bed is held (i.e., temporary discharge). CRE carriers had a 1.8-fold increased risk of readmission within 365 days of discharge. Notably, 8 Orange County nursing homes did not receive patient transfers from other facilities due to lack of interfacility transfer (*n* = 5) or lack of transfer data (*n* = 3).

### CRE spread and control scenarios

Our initial conditions assumed a CRE-naive region (i.e., no CRE cases in any health-care facility or among recently discharged patients) on day 0 and that each nursing home and LTAC's transmission coefficient (β) was calibrated to reach a target prevalence of 25% in LTACs and 8% in nursing homes 7 years from CRE introduction (year 0). We then parameterized the intensive care unit and general ward β coefficients, taking Orange County data into account, so that CRE prevalence trends matched those currently seen in Orange County facilities based upon epidemiologic surveys conducted in year 4 of CRE emergence (16). This corresponded to 75% and 50% of the average nursing home β's for intensive care units and general wards, respectively.

Our initial experimental scenario assumed that no CRE-specific control measures were in place—that is, a facility would detect only those CRE cases incidentally identified from cultures obtained for clinical reasons. As a result, only a fraction of CRE carriers would be detected; we assumed that for every carrier identified incidentally through clinical cultures, 8 would remain undetected. Facilities would place identified CRE carriers on contact precautions (i.e., a single room and use of gloves and gowns by staff). Known carriers would remain on contact precautions when transferred to other facilities or readmitted to the same facility. Nursing home residents with CRE infection (assumed to be 50% of known carriers) were placed in contact precautions for 10 days. Contact precautions reduced transmission by 50%, a combination of the efficacy of the intervention and health-care-worker compliance with the intervention (8).

Other experimental scenarios revolved around the recommendations in the CDC's 2012 CRE toolkit (7). These control strategies implement admission surveillance testing for CRE for patients directly transferred to hospitals or LTACs from another hospital or nursing home, followed by contact precautions (single room, staff glove and gown use) for those testing positive or with a prior history of CRE known to that institution. Testing consisted of rectal screening with a 1-day turnaround time, based upon chromogenic testing, with the test sensitivity and specificity of testing further adjusted for the sensitivity of a single rectal swab compared with multiple or multisite swabs (17–20).

The second experimental scenario represented uncoordinated CRE control, with each individual hospital acting independently in implementing active CRE surveillance and contact isolation when the number of CRE cases in that facility exceeded a certain trigger threshold. This is consistent with the facility-level recommendations in the CRE toolkit. The third scenario represented coordinated regional CRE control in which all hospitals cooperated in a regional CRE containment approach, implementing active CRE surveillance and contact precautions when CRE appeared in a certain number of hospitals (i.e., a trigger threshold) in Orange County. This scenario is consistent with the coordinated regional response recommended in the CRE toolkit.

### Model outcomes and sensitivity analyses

Each simulation experiment involved running the Orange County model 50 times, with each run consisting of 1,000 trajectories for 10–15 simulated years to account for the stochasticity in the model. Reported results are the mean value, median value, and data distribution from each experiment.

Sensitivity analyses varied the trigger threshold for each scenario: for the uncoordinated CRE control scenario, individual hospital trigger thresholds of 1, 10, 20, 50, and 100 CRE cases identified, and for the coordinated regional CRE control scenario, countywide trigger thresholds of 1, 10, and 20 hospitals detecting a CRE case. Additional scenarios also explored varying hospital compliance (range, 15%–50% (16)) with implementing uncoordinated CRE control measures after identifying 10 CRE cases. In each of the aforementioned experiments, the control policy strategy of interest was in place from the time of initial CRE emergence, was instituted as soon as the relevant thresholds were met, and was evaluated over 10 simulated years. An additional set of experiments evaluated the impact of waiting (e.g., 1 or 3 years) until after CRE became endemic (simulation year 7) to implement active CRE surveillance and subsequent coordinated regional CRE control and the 5–7 years following the initiation of these interventions (i.e., a 15-year total time horizon).

## RESULTS

**Figure 1.**
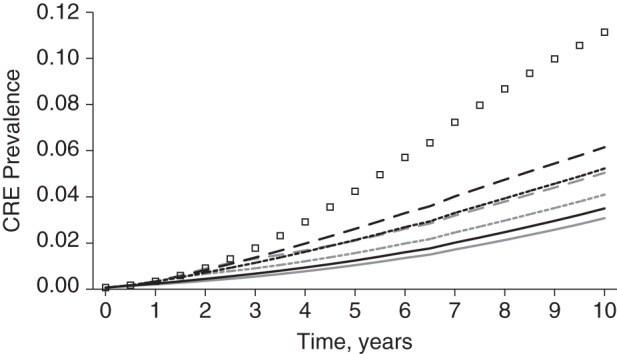
Simulated total countywide prevalence of carbapenem-resistant *Enterobacteriaceae* (CRE) in Orange County, California, in a model with no specific CRE control measures, uncoordinated CRE control measures, and coordinated regional CRE control measures implemented at trigger thresholds of 1, 10, and 20. The line of squares represents no specific control measures; black lines represent uncoordinated control measures at trigger thresholds of 1 (solid line), 10 (short-dashed line), and 20 (long-dashed line); and gray lines represent coordinated regional control at trigger thresholds of 1 (solid line), 10 (short-dashed line), and 20 (long-dashed line).

**Figure 2.**
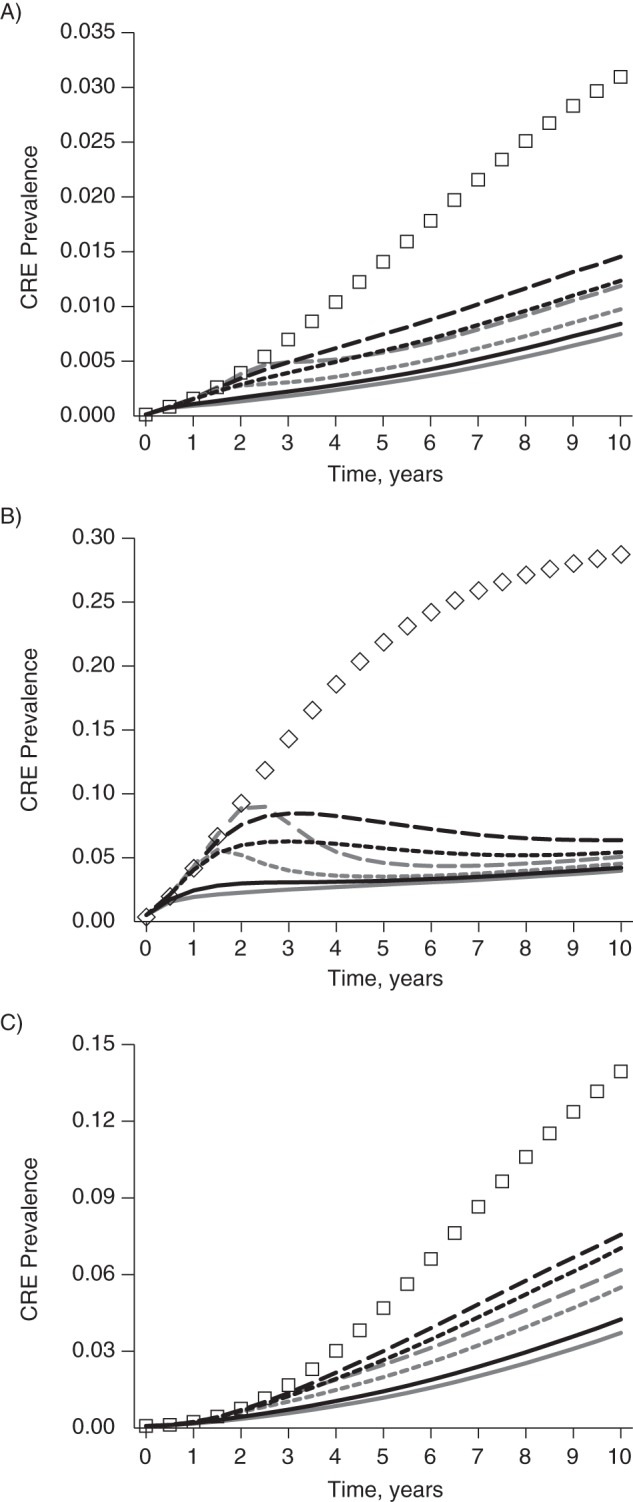
Simulated prevalence of carbapenem-resistant *Enterobacteriaceae* (CRE) in Orange County, California, in a model with no specific CRE control measures, uncoordinated CRE control measures, and coordinated regional CRE control measures implemented at trigger thresholds of 1, 10, and 20, by type of health-care facility. A) acute-care hospitals; B) long-term acute-care hospitals; C) nursing homes. The line of squares represents no specific control measures; black lines represent uncoordinated control measures at trigger thresholds of 1 (solid line), 10 (short-dashed line), and 20 (long-dashed line); and gray lines represent coordinated regional control at trigger thresholds of 1 (solid line), 10 (short-dashed line), and 20 (long-dashed line). (Note that *y*-axis scales are not the same across panels.)

In the absence of specific CRE control measures, CRE prevalence reached a countywide average of 11.1% 10 years after CRE introduction. The line of squares in Figures 1 and 2 shows the estimated CRE prevalence over time in the absence of specific CRE control measures. CRE would rapidly spread throughout the region and reach a prevalence of 3.1% in acute-care hospitals, 28.9% in LTACs, and 13.9% in nursing homes in 10 years (Figure 2). Countywide, 16,495 patients would acquire CRE (both newly acquired and reacquired) within those 10 years.

Figure 1 shows the Orange County CRE prevalence for each scenario and trigger (results for triggers greater than 20 are shown in Web Figure 1, available at http://aje.oxfordjournals.org/). Coordinated regional approaches were consistently more effective than uncoordinated approaches. However, coordinated and uncoordinated approaches yielded similar results when very low triggers were used (trigger of 1, Figure 1). As triggers became higher (e.g., allowing for more cases before implementation of CRE control measures), the advantages of regional approaches grew. For example, uncoordinated approaches with a trigger of 10 cases resulted in a 5.2% countywide CRE prevalence (a 53.0% relative reduction compared with baseline), while a coordinated approach with a trigger of 10 hospitals resulted in a 4.1% countywide CRE prevalence (63.2% relative reduction) at year 10. With a trigger of 20, uncoordinated and coordinated regional approaches led to 45% and 55% relative reductions in countywide prevalence, respectively, at year 10.

Table 2 shows the number of transmission events averted countywide over a 10-year period for both approaches as compared with no CRE control. Over a 5-year period, aggressive control measures (trigger of 1) reduced transmission events by almost 3,000 cases regardless of a coordinated (2,976 events) or uncoordinated (2,789 events) approach. A less aggressive trigger of 10 resulted in 408 more averted cases using the regional coordinated approach as compared with the uncoordinated approach. An even less stringent trigger of 20 also resulted in more averted cases (*n* = 223) using the regional coordinated approach. LTACs had the greatest reduction in transmission events, due to their higher CRE prevalence in our model.

**Table 2.** Average Number of CRE Transmission Events Averted Countywide in Orange County, California, Over a 10-Year Period as Compared With No CRE-Specific Control Measures, for Uncoordinated and Coordinated Regional Approaches Using Trigger Thresholds of 1, 10, and 20

Time Since CRE Emergence, years	Type of Approach and Trigger Threshold					
	Uncoordinated Approaches			Coordinated Regional Approaches		
	1	10	20	1	10	20
1	48	6	1	62	1	1
2	294	121	57	342	115	11
3	815	461	294	905	546	226
4	1,644	1,067	768	1,780	1,298	809
5	2,789	1,953	1,497	2,976	2,361	1,720
6	4,233	3,108	2,473	4,480	3,715	2,914
7	5,951	4,506	3,678	6,268	5,331	4,357
8	7,902	6,107	5,074	8,297	7,168	6,003
9	10,030	7,861	6,616	10,513	9,170	7,804
10	12,283	9,721	8,260	12,862	11,290	9,712

Abbreviation: CRE, carbapenem-resistant *Enterobacteriaceae*.

Figure 2 shows CRE prevalence for each facility type. Both uncoordinated and coordinated regional approaches showed the greatest benefit in LTACs, followed by nursing homes. Coordinated regional approaches produced a more rapid decline in CRE prevalence than did the uncoordinated strategy. The effect increased with higher trigger thresholds, especially in LTACs (Figure 2B). Web Table 1 quantifies the impact of CRE interventions on acute-care hospitals, LTACs, and nursing homes for each trigger up to 20. Both strategies generated a statistically significant difference in prevalence from baseline after 2 years with a trigger of either 1 or 10. Strategies with a trigger of 20 required at least 3 years to show a statistically significant difference from baseline.

Compared with a trigger of 1, delaying control until a trigger of 10 was reached resulted in an approximate 30% loss of effect for uncoordinated strategies and an approximate 20% loss of effect for coordinated strategies in acute-care hospitals and LTACs by year 5 (Web Table 1). Reductions in impact for a trigger of 10 versus a trigger of 1 were even greater (44% for uncoordinated and 30% for coordinated) for nursing homes. Delaying until a trigger of 20 was reached resulted in approximately half the effect by year 5 compared with a trigger of 1 (an approximate 50% loss for acute-care hospitals, 40% loss for LTACs, and a 60% loss for nursing homes via either strategy; Web Table 1).

**Figure 3.**
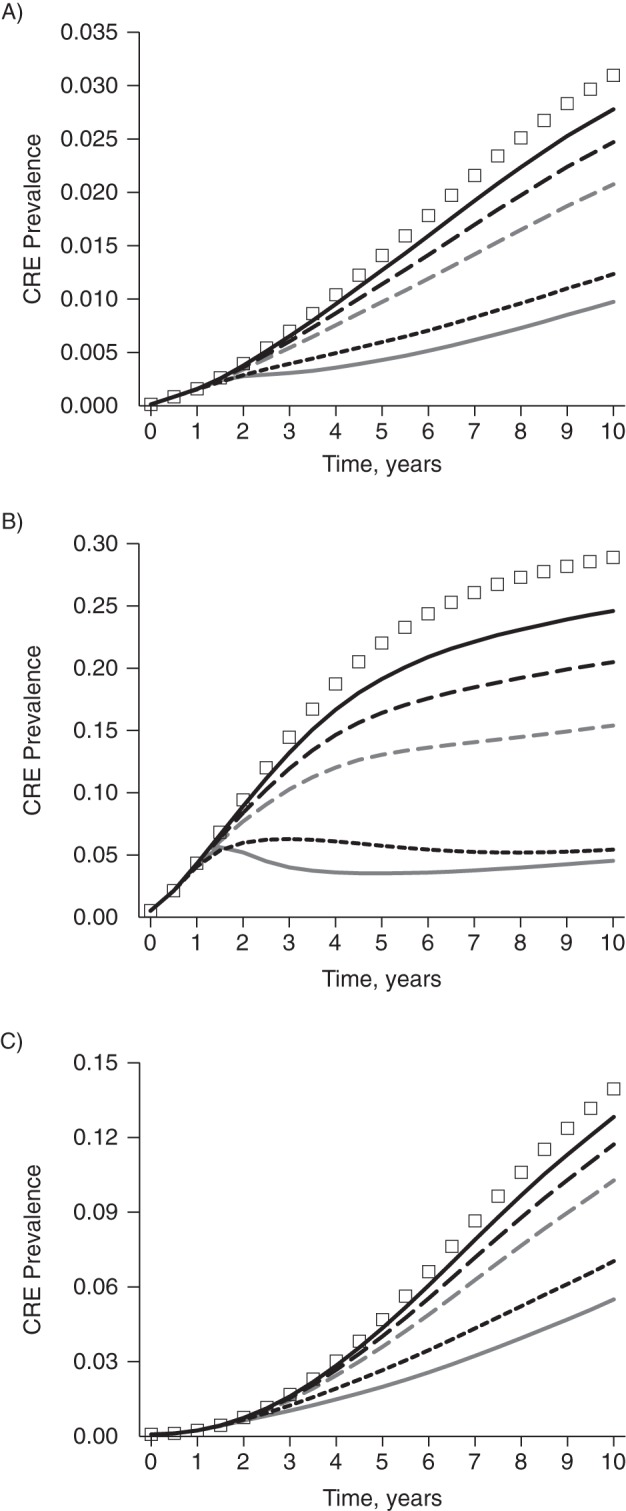
Simulated impact of hospital compliance with modeled control measures on the prevalence of carbapenem-resistant *Enterobacteriaceae* (CRE) in Orange County, California, when 15% (solid black line), 30% (long-dashed black line), 50% (dashed gray line), and 100% (short-dashed black line) of hospitals implement uncoordinated CRE control measures at a trigger threshold of 10, by type of health-care facility. A) acute-care hospitals; B) long-term acute-care hospitals; C) nursing homes. The line of squares represents no specific control measures, while the solid gray line represents coordinated regional control at a trigger of 10. (Note that *y*-axis scales are not the same across panels.)

Compliance with uncoordinated approaches substantially affected the benefits of CRE control measures (Figure 3). The countywide CRE prevalence reached 10.1% at year 10 when only 15% of hospitals implemented active surveillance at detection of 10 cases (an 8.8% relative reduction over those 10 years).

**Figure 4.**
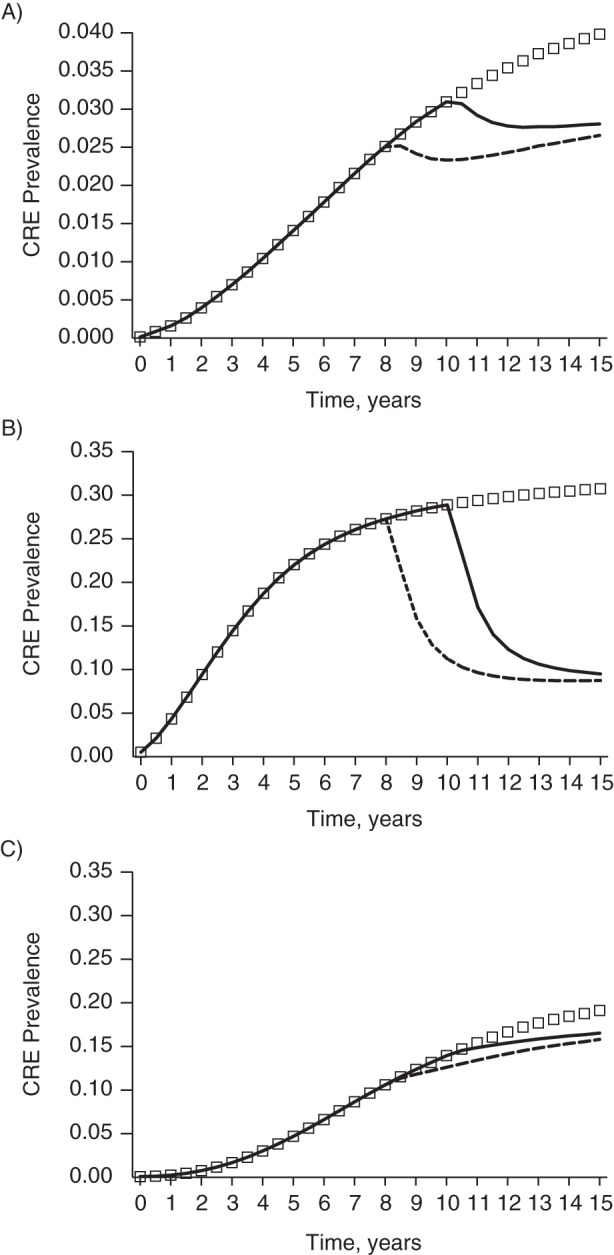
Simulated total countywide prevalence of carbapenem-resistant *Enterobacteriaceae* (CRE) in Orange County, California, in a model with no specific CRE control measures and coordinated regional CRE control measures implemented once CRE has become endemic (years 8 and 10). The line of squares represents no specific control measures; black lines represent coordinated regional CRE control implemented in year 8 (dashed line) or year 10 (solid line). (Note that *y*-axis scales are not the same across panels.)

Figure 4 shows what would happen if coordinated regional CRE control measures were not implemented until CRE became endemic in Orange County. If CRE is left unchecked without specific control measures, countywide CRE prevalence reaches 14.9% at year 15. If coordinated regional approaches are implemented in year 8, the countywide prevalence decreases to 9.4% by year 10 but steadily increases to 11.6% by year 15. If coordinated approaches are delayed until year 10, they have a marginal impact on CRE prevalence (12.2% at year 15). LTACs experience the most gain with late implementation, but the levels to which CRE are reduced are not the same as when implementation occurs before CRE become endemic (approximately 9% vs. approximately 5%; Figure 4B vs. Figure 2B).

## DISCUSSION

Based on the current epidemiologic trends in CRE spread, our simulation model suggests that without implementation of CDC-recommended interventions, CRE will continue to spread among health-care facilities until they become endemic in Orange County. LTACs, which are well known epidemiologically as focal points for concentration of CRE (21–23), would experience by far the highest increase in prevalence. Likewise, nursing homes would experience a higher resulting prevalence, compared with the much smaller increases seen in hospitals. The effects observed for LTACs are due to their having greater importation of CRE among admitted patients, generally higher transmission coefficients (calibrated on the basis of studies in the literature (23–26)), longer patient lengths of stay, smaller sizes (e.g., lower bed capacity), and substantial interconnectivity with other facilities. The effects in nursing homes are due to their higher transmission coefficients (calibrated on the basis of studies in the literature (27)), longer lengths of stay, and extensive mixing among patients.

One way of mitigating CRE spread is for each acute-care hospital and LTAC to initiate CRE screening of all transfer patients (including resultant contact precautions for carriers) as soon as the number of CRE cases in that facility exceeds a certain threshold. The key is to set a low threshold (1, 10, and 20 CRE cases) for implementing control measures. If the threshold is too high, if the facility does not act quickly enough, or if the facility is not sufficiently compliant in implementing control measures, then CRE spread may not be adequately abated. Once a sufficient number of CRE cases are percolating throughout the network of facilities in a region, control becomes considerably more difficult. A major drawback of relying on individual facilities to detect cases and implement measures accordingly is the possibility that not all facilities would have the same determination of an important threshold or the same ability to ensure compliance. For instance, an individual facility might not be able to catch cases in a timely manner or might not be compliant with implementing control measures.

By comparison, we show that bringing facilities together to collectively signal the countywide presence of CRE cases and implement regional control measures can be substantially be more effective in stemming the spread of CRE. As the individual trigger for action is raised, the regional impact becomes increasingly greater than the sum of its parts. Diffusing the responsibility of detecting CRE cases across facilities makes it more likely that the CRE's spread will be detected earlier and thus contained before percolating extensively throughout the network. Countywide monitoring gives more leeway to catch the spread of CRE early. In other words, a facility does not have to wait until it sees CRE to implement CRE control measures. Therefore, the countywide triggers do not have to be as low to still be substantially more effective at controlling CRE than facilities acting individually. However, this assumes that hospitals are willing to be responsive to CRE cases occurring elsewhere in the county.

Coordinating facilities in a region may entail setting up an information system or implementing other means to facilitate more rapid communications among facilities and a centralized authority (e.g., a local or state public health department) to initiate and monitor infection control measures. Such a regional approach could have secondary benefits, such as engendering camaraderie among health-care facilities, helping control other pathogens, and fostering exchange of information and experience on disease control. A key consideration is that the burden of screening would be disproportionately shouldered by hospitals and LTACs, while the benefit would be greatest to LTACs and nursing homes. Therefore, coordinating authorities must be sensitive to this dynamic and help all facilities understand the collective and long-term benefits of such coordination.

Our results showed that impact of uncoordinated and coordinated regional approaches was, not unexpectedly, greatest in LTACs. This was probably due to many factors, including LTACs' higher prevalence rate based upon epidemiologic data, the aggressive control measures adopted by hospitals (including LTACs) as compared with nursing homes, and the high interconnectedness of LTACs with other facilities (including frequent patient-sharing with hospitals also implementing control measures). The relatively small size of LTACs also led to faster relative reductions in prevalence, as 1 fewer CRE case had a larger effect on the total prevalence.

Our simulation experiments for both uncoordinated and coordinated approaches modeled highly proactive infection control strategies. We demonstrated that preventing the spread of CRE before it fully manifests is much more effective than waiting for the problem to fully declare itself. Nevertheless, this requires hospitals to be willing to act before an outbreak or widespread transmission is noted, which may require a change of culture for many institutions. Once CRE has become endemic, more aggressive bundled interventions may be necessary. In a recent study, Hayden et al. (28) determined that a bundled intervention including high compliance screening, contact isolation, and decolonization with daily chlorhexidine bathing led to statistically significant reductions in *Klebsiella pneumoniae* carbapenemase-producing *Enterobacteriaceae* acquisition, prevalence, and bloodstream infections in LTACs.

Our results support the CDC's efforts to engage local and state health departments in active strategies focused on controlling the spread of antimicrobial-resistant pathogens (14). The extensive patient-sharing among health-care facilities in a region is the basis for the importance of interfacility cooperation (8, 29). Now evidence from both simulation modeling and clinical studies supports cooperation among facilities when attempting to control the spread of MRSA (8) and vancomycin-resistant enterococci (9, 10) in the United States and CRE in Israel (30, 31).

Our study does have limitations. All models are simplifications of real life (32), and as such they cannot represent every possible outcome. With our model, we attempted to portray outcomes that would be reasonably likely if CRE control strategies were widely adopted across a region. However, we assumed fairly proactive and aggressive measures. In several scenarios, we assumed that all hospitals would reliably implement CRE control strategies once the triggers had been reached, presumably in response to a regional public health request; in reality, individual facilities may not implement any control strategy, and consistency and sustainability may be an issue. Our results are also limited by the fact that the model was tailored to 1 region's extensive data on patient-sharing across facilities. To the extent that patient-sharing is uncommon due to large geographic distances between facilities or insurance restrictions, results may be quite different in different parts of the country. Moreover, our model assumed minimal community transmission of CRE and did not include pediatric facilities, although the literature suggests that children are less important drivers of the current epidemic. Finally, our model does not currently provide the necessary detail with which to evaluate high-risk patient characteristics, such as the impact of ventilator dependency or other comorbidity in driving CRE transmission.

Our study suggests that CRE will become endemic in a region without adequate interventions. Individual facilities can substantially reduce the spread of CRE by implementing the recommendations in the CDC toolkit (7), particularly if they initiate such control measures before CRE has become endemic. The most effective strategy may be to employ a regional coordinated approach in which all facilities implement the CDC toolkit measures when CRE cases appear in a threshold number of facilities. This proactive and coordinated approach appears to rapidly confine the spread of CRE.

## Supplementary Material

Web Material
