# Supplementary material for: The Potential Trajectory of Carbapenem-Resistant Enterobacteriaceae, an Emerging Threat to Health-Care Facilities, and the Impact of the Centers for Disease Control and Prevention Toolkit
Source: Am J Epidemiol. 2016 Feb 8;183(5):471–9. doi: 10.1093/aje/kwv299 (PMC4772438; doi:10.1093/aje/kwv299)
Supplement: Web Material [file supp_183_5_471__index.html]

The Potential Trajectory of Carbapenem-Resistant Enterobacteriaceae, an Emerging Threat to Health-Care Facilities, and the Impact of the Centers for Disease Control and Prevention Toolkit — Web Material 

# The Potential Trajectory of Carbapenem-Resistant *Enterobacteriaceae*, an Emerging Threat to Health-Care Facilities, and the Impact of the Centers for Disease Control and Prevention Toolkit

## Web Material

Web Material

- Web Material - Pdf file
