# Supplementary material for: The Potential Trajectory of Carbapenem-Resistant Enterobacteriaceae, an Emerging Threat to Health-Care Facilities, and the Impact of the Centers for Disease Control and Prevention Toolkit
Source: Am J Epidemiol. 2016 Feb 8;183(5):471–9. doi: 10.1093/aje/kwv299 (PMC4772438; doi:10.1093/aje/kwv299)
Supplement: Web Material [file supp_kwv299_kwv299supp.pdf]

Web Figure 1. Prevalence of carbapenem-resistant *Enterobacteriaceae* (CRE), by facility type, for uncoordinated control measures implemented at trigger thresholds of 20 (short-dashed black line), 50 (solid black line), and 100 (long-dashed black line) as compared with no specific control measures (gray line) in A) acute-care hospitals, B) long-term acute-care hospitals (LTACs), and C) nursing homes.

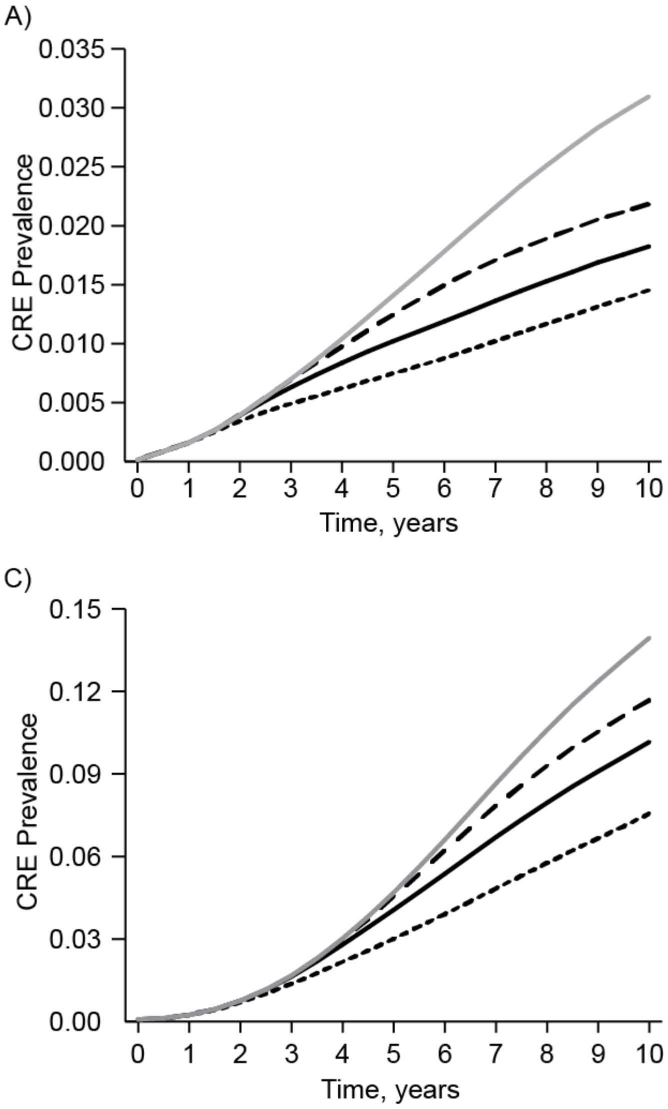

Web Table 1. Reduction in the prevalence of carbapenem-resistant *Enterobacteriaceae* (CRE) (95% CI in difference), by facility type, in comparison with baseline over a 10-year period for uncoordinated and coordinated regional approaches

Approach	Trigger	Year(s)									
		1	2	3	4	5	6	7	8	9	10
**Acute-Care Hospitals**											
Uncoordinated	1	0.02 (0.01, 0.02)	0.08 (0.08, 0.08)	0.17 (0.17, 0.17)	0.28 (0.28, 0.29)	0.41 (0.41, 0.41)	0.55 (0.54, 0.56)	0.68 (0.68, 0.69)	0.82 (0.82, 0.82)	0.95 (0.95, 0.96)	1.08 (1.08, 1.09)
	10	0.00 (0.00, 0.00)	0.03 (0.02, 0.03)	0.09 (0.09, 0.09)	0.17 (0.17, 0.17)	0.28 (0.27, 0.28)	0.39 (0.39, 0.39)	0.51 (0.50, 0.51)	0.62 (0.62, 0.63)	0.74 (0.74, 0.74)	0.85 (0.84, 0.85)
	20	0.00 (0.00, 0.00)	0.01 (0.01, 0.01)	0.05 (0.05, 0.05)	0.12 (0.12, 0.12)	0.20 (0.20, 0.21)	0.30 (0.30, 0.30)	0.41 (0.40, 0.41)	0.51 (0.51, 0.51)	0.62 (0.61, 0.62)	0.71 (0.71, 0.71)
Coordinated regional	1	0.02 (0.02, 0.02)	0.09 (0.09, 0.09)	0.19 (0.19, 0.20)	0.31 (0.31, 0.31)	0.44 (0.44, 0.45)	0.58 (0.58, 0.58)	0.72 (0.72, 0.73)	0.87 (0.86, 0.87)	1.00 (1.00, 1.00)	1.13 (1.13, 1.13)
	10	0.00 (0.00, 0.00)	0.02 (0.02, 0.02)	0.10 (0.10, 0.10)	0.21 (0.21, 0.21)	0.34 (0.34, 0.34)	0.47 (0.47, 0.47)	0.61 (0.60, 0.61)	0.74 (0.74, 0.74)	0.87 (0.87, 0.87)	0.99 (0.99, 0.99)
	20	0.00 (0.00, 0.00)	0.00 (0.00, 0.00)	0.03 (0.03, 0.03)	0.12 (0.12, 0.12)	0.23 (0.23, 0.24)	0.36 (0.36, 0.36)	0.49 (0.49, 0.49)	0.61 (0.61, 0.61)	0.73 (0.73, 0.73)	0.84 (0.84, 0.85)
**Long-Term Acute-Care Hospitals (LTACs)**											
Uncoordinated	1	0.67 (0.65, 0.68)	2.46 (2.44, 2.48)	4.69 (4.66, 4.71)	6.95 (6.93, 6.98)	9.05 (9.03, 9.08)	10.89 (10.87, 10.92)	12.47 (12.44, 12.49)	13.80 (13.78, 13.82)	14.93 (14.91, 14.95)	15.88 (15.87, 15.90)
	10	0.05 (0.04, 0.07)	0.90 (0.87, 0.92)	2.6 (2.57, 2.62)	4.61 (4.58, 4.64)	6.63 (6.60, 6.66)	8.49 (8.46, 8.52)	10.14 (10.11, 10.16)	11.56 (11.54, 11.59)	12.79 (12.77, 12.81)	13.83 (13.81, 13.85)
	20	0.01 (-0.01, 0.03)	0.37 (0.35, 0.40)	1.58 (1.55, 1.61)	3.30 (3.27, 3.33)	5.16 (5.13, 5.19)	6.95 (6.92, 6.98)	8.58 (8.55, 8.60)	10.02 (10.00, 10.05)	11.28 (11.26, 11.30)	12.37 (12.35, 12.39)
Coordinated regional	1	0.89 (0.88, 0.91)	2.91 (2.89, 2.93)	5.2 (5.18, 5.23)	7.46 (7.43, 7.49)	9.53 (9.50, 9.56)	11.34 (11.31, 22.36)	12.88 (12.86, 12.90)	14.19 (14.17, 14.22)	15.30 (15.28, 15.32)	16.24 (16.23, 16.26)
	10	0.01 (-0.01, 0.02)	0.86 (0.83, 0.88)	3.14 (3.12, 3.17)	5.63 (5.61, 5.66)	7.92 (7.90, 7.95)	9.90 (9.88, 9.93)	11.58 (11.56, 11.60)	12.99 (12.97, 13.02)	14.18 (14.16, 14.20)	15.18 (15.16, 15.20)
	20	0.01 (0.00, 0.03)	0.07 (0.04, 0.09)	1.21 (1.18, 1.24)	3.53 (3.50, 3.56)	5.97 (5.94, 5.99)	8.12 (8.10, 8.15)	9.96 (9.94, 9.98)	11.50 (12.77, 12.82)	12.79 (12.77, 12.82)	13.88 (13.86, 13.90)

Nursing Homes											
Uncoordinated	1	0.02 (0.02, 0.02)	0.10 (0.09, 0.10)	0.27 (0.27, 0.28)	0.57 (0.56, 0.57)	0.98 (0.97, 0.98)	1.47 (1.46, 1.47)	2.03 (2.02, 2.04)	2.63 (2.62, 2.64)	3.23 (3.22, 3.24)	3.80 (3.79, 3.81)
	10	0.01 (0.01, 0.01)	0.03 (0.03, 0.03)	0.11 (0.11, 0.12)	0.29 (0.28, 0.29)	0.55 (0.55, 0.56)	0.91 (0.90, 0.92)	1.33 (1.32, 1.34)	1.78 (1.78, 1.79)	2.25 (2.24, 2.26)	2.69 (2.68, 2.70)
	20	0.01 (0.01, 0.01)	0.02 (0.02, 0.02)	0.06 (0.06, 0.06)	0.17 (0.17, 0.18)	0.37 (0.36, 0.38)	0.65 (0.64, 0.65)	0.98 (0.97, 0.99)	1.36 (1.35, 1.37)	1.74 (1.73, 1.75)	2.12 (2.10, 2.13)
Coordinated regional	1	0.03 (0.03, 0.03)	0.13 (0.12, 0.13)	0.33 (0.33, 0.33)	0.65 (0.65, 0.66)	1.09 (0.08, 1.09)	1.62 (1.61, 1.62)s	2.21 (2.20, 2.21)	2.84 (2.83, 2.85)	3.48 (3.47, 3.48)	4.08 (4.07, 4.09)
	10	0.01 (0.01, 0.01)	0.04 (0.03, 0.04)	0.16 (0.15, 0.16)	0.40 (0.40, 0.41)	0.76 (0.75, 0.77)	1.21 (1.21, 1.22)	1.73 (1.72, 1.74)	2.29 (2.28, 2.30)	2.84 (2.83, 2.85)	3.38 (3.37, 3.39)
	20	0.01 (0.01, 0.01)	0.02 (0.01, 0.02)	0.06 (0.06, 0.06)	0.21 (0.21, 0.21)	0.48 (0.48, 0.49)	0.85 (0.84, 0.86)	1.29 (1.28, 1.30)	1.77 (1.76, 1.78)	2.25 (2.24, 2.26)	2.72 (2.71, 2.73)
